# Investigation of clinical features of dysgraphia related to the subtypes of developmental coordination disorder in children regarding high IQ

**DOI:** 10.1192/j.eurpsy.2021.580

**Published:** 2021-08-13

**Authors:** S. Hamdioui, L. Vaivre-Douret

**Affiliations:** 1 Université De Paris, Faculty of Society and Humanity, Division of Psychology, Paris, France; 2 University Of Paris-saclay, Uvsq, National Institute of health and Medical Research (INSERM UMR 1018-CESP), Faculty of Medicine, Villejuif, France; 3 Department Of Endocrinology, Imagine Institute, Necker-Enfants Malades University Hospital, Paris, France; 4 Université De Paris, Faculty of Health, Division of Medicine Paris Descartes, Paris, France; 5 (institut Universitaire De France, Iuf), University Institute of France, Paris, France; 6 Necker-enfants Malades, University Hospital, Department of Child Psychiatry, Assistance Publique-Hôpitaux de Paris (AP-HP. Centre), Paris, France

**Keywords:** Dysgraphia, Neurovisual impairments, Developmental coordination disorder subtypes, Intellectual quotient

## Abstract

**Introduction:**

Handwriting disorder is commonly observed in Developmental Coordination Disorder (DCD) (87-88%) and is often noted in children with high Intellectual Quotient (HIQ).Two mainly pure DCD subtypes: ideomotor-DCD (IM), visuospatial/or visuoconstructional-DCD (VSC) and a mixed subtype (MX) were identified in the literature but nothing is known regarding IQ and dysgraphia.

**Objectives:**

To refine the specific clinical features of dysgraphia related to DCD subtypes regarding IQ levels.

**Methods:**

Neurovisual, neuropsychological, neuropsychomotor functions, and handwriting performances of 38 children (6-to-12 years-old: mean 9y, SD 2.7) diagnosed with DCD (DSM-5 criteria) were collected. Two matched groups were analyzed according to their IQ: 19 (TC) typical children (IQ=90-110) and 19 HIQ children (IQ> 120).

**Results:**

IQ scores were not significantly associated with dysgraphia. There isa significant difference between TC vs HIQ with a lower rate of IM-DCD respectively 11% vs 5% (p=.035) and 68% vs 37% for VSC-DCD (p=.03) but 21% vs 58% in MX-DCD (p=.41). Dysgraphia was significantly more present in TC group with MX-DCD and in HIQ with VSC-DCD. A negative correlation between Kho’s’ cubes test failure (p=.006), visual-spatial memory (p=.05) and VSC-DCD was noted in HIQ group. The deficit of visual spatial memory was significantly related to dysgraphia in HIQ children (p=.01) associated to visual gnosis impairment (p=.03).

**Conclusions:**

Dysgraphia was significantly found with VSC-DCD subgroup in FIQ>120 with specific features of visual perception disorders suggesting more involvement of the right cortex. These results suggest that VSC-DCD in HIQ could be a neurovisual impairment rather than a pure VSC-DCD.

